# Factors Associated with Poststroke Anxiety: A Systematic Review and Meta-Analysis

**DOI:** 10.1155/2017/2124743

**Published:** 2017-02-22

**Authors:** Francesca Wright, Simiao Wu, Ho-Yan Yvonne Chun, Gillian Mead

**Affiliations:** ^1^University of Edinburgh, Edinburgh, UK; ^2^Centre for Clinical Brain Sciences, University of Edinburgh, Edinburgh, UK; ^3^Department of Geriatric Medicine, University of Edinburgh, Edinburgh, UK; ^4^Centre for Cognitive Ageing and Cognitive Epidemiology, Edinburgh, UK

## Abstract

*Background and Purpose.* Anxiety affects 25% of stroke survivors. There are no effective treatments. Poststroke depression, prestroke anxiety and depression, locus of control, coping, confidence, fatigue, and sleep are factors that may be associated with poststroke anxiety and can potentially be targeted by therapy. We systematically reviewed the literature and performed a meta-analysis to identify associations with these factors.* Methods*. We searched electronic databases from January 2014 to July 2015 to complement a literature search performed from inception to May 2014. Study quality was assessed using an internationally endorsed checklist. We used odds ratios (ORs) to estimate the strength of associations and random-effects modelling to calculate summary effect sizes.* Results.* There were 24 studies recruiting 15448 patients. Quality of reporting was satisfactory. 13 studies with 2408 patients reported associations between poststroke anxiety and poststroke depression (OR = 4.66, 95% confidence interval: 2.23, 9.74). One study reported association with prestroke anxiety, three with prestroke depression, one with fatigue, and two with sleep. No studies reported on locus of control, coping, or confidence.* Conclusion.* Poststroke anxiety was associated with depression but there are limited data on other modifiable associations. Further research is needed to identify potential targets for treatment.

## 1. Introduction

A clinically significant mental health disorder occurs in greater than one-third of poststroke patients and may be associated with increased morbidity and mortality [1]. The most common disorders after stroke are depression and anxiety [2]. Worldwide, anxiety disorders are the most prevalent group of mental health disorders, with an estimated lifetime prevalence of approximately 11% [3]. Anxiety following a stroke or transient ischaemic attack (TIA) occurs in about 24% of patients [4] and is a distressing problem associated with poorer health related quality of life [5]. Despite its prevalence, poststroke anxiety (PSA) is an understudied area in comparison to other psychological poststroke disorders such as depression [4]. There are no studies on the prevention of PSA [2] and very few randomised trials of intervention to treat it [6]. This may partly reflect a lack of data reporting its risk factors and associations. In order to develop effective therapies for PSA, we need to better understand its associations which could provide targets for treatment.

Although there has been a previous systematic review on associations of PSA [7], a meta-analysis of the strength of associations was not performed. Furthermore the searches were performed in May 2014 and further studies have been published since. In our systematic review we wish to focus on potentially modifiable factors that could be targeted by therapy. We will determine the association with the following factors of interest: poststroke depression (PSD), prestroke anxiety and depression, locus of control, coping, confidence, fatigue, and sleep, and we will perform a meta-analysis to estimate the strength of associations.

## 2. Methods

Our review updates and complements an earlier systematic review on the associations of PSA by Menlove et al. which reported searches from inception to May 2014. Their search strategy can be found in their primary publication [7].

We applied our search strategy from Jan 2014 to 19th July 2015 using the databases MEDLINE, EMBASE, CINAHL PLUS, AMED, PsychINFO, and ProQuest dissertation. There was an overlap in the search period (four months) with Menlove's review to ensure studies that were in print were not missed. We used search terms synonymous with stroke and anxiety obtained from a Cochrane review of interventions for PSA [6]. This review's search strategy can be found in the Supplementary Material available online at https://doi.org/10.1155/2017/2124743.

One author (FW) screened all titles and excluded the obviously irrelevant. The remaining citations were exported to EndNote and two authors (FW and HYC) independently screened the abstracts to decide which to obtain as a full text. The two authors independently read all full texts and decided which studies fulfilled the eligibility criteria. Any discrepancies were dealt with through discussion.

Our inclusion criteria were as follows: (1) studies on stroke patients including ischaemic, haemorrhagic, or unspecified stroke subtype; (2) incidence studies, cohort studies, cross-sectional studies, case control studies, or case series that made use of consecutive patient recruitment within clearly defined geographical and time-limited boundaries; (3) assessed anxiety using a validated anxiety scale or clinical diagnostic criteria in a psychiatric interview; (4) used regression analyses; and (5) reported in English.

Studies were excluded if they (1) had mixed populations (unless separate results for stroke patients were reported); (2) were limited to select patient characteristics (such as age, gender, and lesion side); (3) used retrospective recruitment or reporting of mood; (4) did not measure anxiety specifically; (5) contained insufficient data for reporting of associations; (6) included participants <18 years old; (7) were case reports or included <10 participants; (8) were intervention studies; or (9) included >25% of participants with TIA rather than stroke.

Two authors (FW and HYC) independently extracted data onto electronic tables. Variables included demographics, time points, and measures for assessing anxiety, proportion who had PSA, statistical methods for analysing associations, and factors including PSD, prestroke anxiety, prestroke depression, locus of control, coping, confidence, fatigue, and sleep disturbance.

The quality of each study was assessed independently by two authors (FW and HYC) using The Strengthening the Reporting of Observational studies in Epidemiology (STROBE) checklist [8] consisting of 22 items required for good reporting of observational studies.

We included studies [9–11] that solely included patients with posttraumatic stress disorder (PTSD) following a stroke as it is classified as an anxiety disorder under the Diagnostic and Statistical Manual of Mental Disorders IV (DSM-IV) criteria and its older versions. While the latest DSM-V no longer includes PTSD under the classification of anxiety disorders [12] all of the included studies used measures based on the DSM-IV-TR or earlier versions.

The authors conducted this systematic review within the Centre for Cognitive Ageing and Cognitive Epidemiology (CCACE) which is funded by the Medical Research Council (MRC) and the Biotechnology and Biological Sciences Research Council (BBSRC).

### 2.1. Statistical Analysis

In order to carry out a meta-analysis of the association between PSA and the factors of interest, we calculated odds ratios (ORs) and 95% confidence intervals (95% CI) from raw data where possible. For meta-analysis, if raw data were not available, ORs and 95% CIs reported in the studies were used. If only correlation coefficients were available, these were converted into ORs using conventional methods [13]. We used funnel plotting to assess publication bias [14].

Random-effects modelling was used to determine the summary estimate of OR [15]. The Cochran *Q* statistic was used to assess heterogeneity between studies, where a *p* value of < 0.05 indicates significant heterogeneity [16]. We partitioned heterogeneity between studies reporting adjusted ORs and those reporting unadjusted ORs [16].

## 3. Results

**Figure 1:**
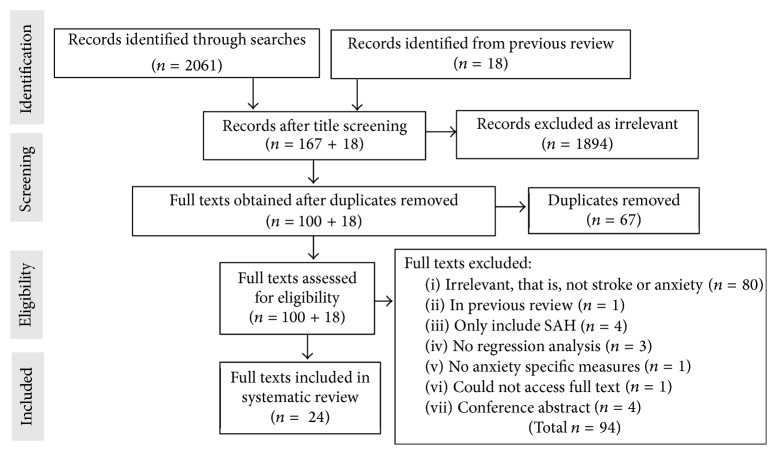
Flow diagram showing study selection process.

Our searches identified 2061 studies. We obtained 100 full texts and six [11, 17–21] fulfilled our eligibility criteria in addition to the 18 studies [9, 10, 22–37] from a previous review [7] which had the same inclusion criteria applied.  Figure 1 shows reasons for exclusion. We included a total of 24 studies.

The 24 studies recruited a total of 15448 participants, with a mean age ranging from 51.7 to 75.2 years (though four studies did not report mean age [20, 22, 25, 34]). The proportion of females ranged from 20.8% to 59.3% with one study not reporting this data [22]. Six studies had participants with ischaemic stroke [25, 26, 29, 31, 32, 36], nine had both ischaemic and haemorrhagic stroke [18, 19, 21, 23, 27, 28, 30, 35, 37], and nine did not specify the type of stroke [9–11, 17, 20, 22, 24, 33, 34]. 13 studies recruited participants from hospital [9, 10, 21, 25–31, 34–36], four from rehabilitation centres [18, 23, 33, 37], three from the community (including outpatient clinics) [11, 19, 20], and four from the population (such as stroke registers) [17, 22, 24, 32]. Studies assessed anxiety at varying time points after stroke, ranging from 3 days to yearly for 20 years.

To assess the presence of anxiety, 13 studies used the Hospital Anxiety and Depression Scale (HADS-A) [17–19, 21, 22, 24, 26, 28, 29, 33–36], three studies used the Hamilton Anxiety Scale (HAM-A) [25, 27, 31], one used the Beck Anxiety Inventory (BAI) [23], two used the DSM-IV criteria [10, 32], one used the Irritability Depression and Anxiety scale (IDA) [30], one used the two-item Generalized Anxiety Disorder questionnaire (GAD-2) [20], two used the Posttraumatic Diagnostic Scale (PDS) [9, 37], and one used the PTSD Checklist Specific for a stressor (PCL-S) [11]. The percentage of participants deemed to have PSA using these scales ranged from 6.06% to 56.4%. The mean frequency of PSA symptoms at baseline was 33.5%.

A range of cut-offs were used in the thirteen studies that measured anxiety using HADS-A. Seven used a cut-off of ≥8 [21, 22, 26, 29, 33, 35, 36], one study used a cut-off of ≥4 [18], one study used a cut-off of ≥5 [28], two studies used a score of 8–10 as possible caseness and a score of ≥11 as probable caseness [17, 24] and two did not specify a cut-off [19, 34].

Sixteen studies excluded patients with moderate or severe cognitive or communication impairment, including aphasia [9–11, 18, 19, 21–23, 26, 28, 29, 31, 34–37].

The quality of studies is summarised in Supplemental Table I. According to the STROBE checklist the background and aims of the majority were clear, and the discussion was satisfactory, with weaknesses mainly in the methodology: two out of 24 studies provided sample size calculations; three explained how missing data were addressed; six indicated the number of participants with missing data for each variable of interest; two described some efforts to address potential sources of bias; and three out of 11 cohort studies described how loss to follow-up was addressed. Ten studies gave sources of funding.

We were only able to carry out meta-analysis on the association of PSA with PSD as the other factors did not have sufficient data.

### 3.1. Association with Poststroke Depressive Symptoms

Thirteen studies (*n* = 2408) reported the association between PSD and PSA. Of which, three studies [21, 32, 36] reported a significant correlation on multivariate analysis and 10 on univariate analysis [9–11, 18, 19, 23, 30, 31, 34, 35]. We found one additional study that did not report the association but provided enough data for us to calculate an OR from which we found a significant association (OR = 8.6, 95% CI 4.46–16.58, *p* ≤ 0.0001) [33]. Another study reported the association between PSA and depression treatment and found a significant correlation on univariate analysis at both 3 and 5 years [22].

#### 3.1.1. Overall Meta-Analysis

**Figure 2:**
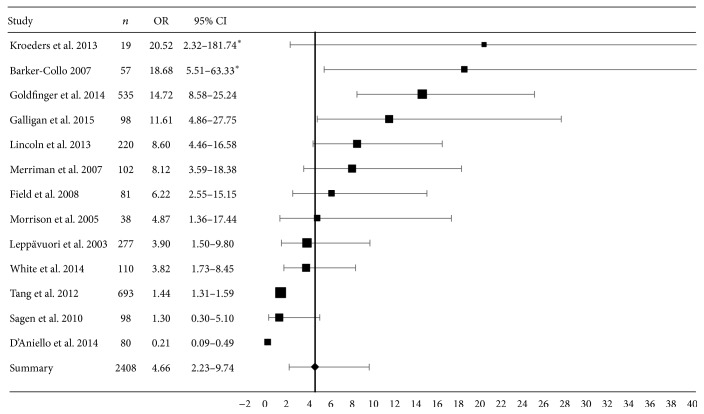
Random-effects meta-analysis for the association between poststroke anxiety and depressive symptoms. Horizontal axis represents the odds ratio (OR) comparing the occurrence of depressive symptoms in patients with and without poststroke anxiety. Horizontal error bars represent the 95% confidence interval (95% CI) of the OR from individual studies. The vertical line represents the summary OR. Symbol size represents the natural log of the number of participants in that study. ^*∗*^The upper limit of the 95% CI beyond 40 does not show in the plot.

Of the 13 studies which had reported the association between PSD and PSA and the additional study from which we were able to calculate an OR, 13 [9–11, 18, 19, 21, 23, 30, 32–36] provided data for ORs and thus were included in the meta-analysis. ORs were provided in four studies [21, 32, 35, 36]; we converted correlation coefficients to ORs in seven [9, 10, 18, 19, 23, 30, 34] and we calculated ORs from raw data in two [11, 33]. The summary estimate of OR for this association was 4.66 (95% CI 2.23–9.74, Figure 2). There was no significant heterogeneity between studies (*Q* = 10.67; degree  of  freedom = 12; *p* = 0.56).

#### 3.1.2. Stratified Meta-Analysis

Three studies reported adjusted ORs [21, 32, 36] for age, sex, modified Rankin score, multidimensional scale of perceived social support, Adelaide activities profile, past depression, and the presence of right frontal acute infarcts. The remaining 10 studies reported unadjusted ORs [9–11, 18, 19, 23, 30, 33–35]. This grouping did not account for a significant part of the heterogeneity between studies (*Q* = 0.12; degree of freedom = 1; *p* = 0.73). The summary OR for the unadjusted studies (*n* = 1344; OR = 5.58; 95% CI 2.33–13.36) was higher (*p* ≤ 0.01) than for the adjusted studies (*n* = 1080; OR = 2.52; 95% CI 1.14–5.61).

#### 3.1.3. Publication Bias

**Figure 3:**
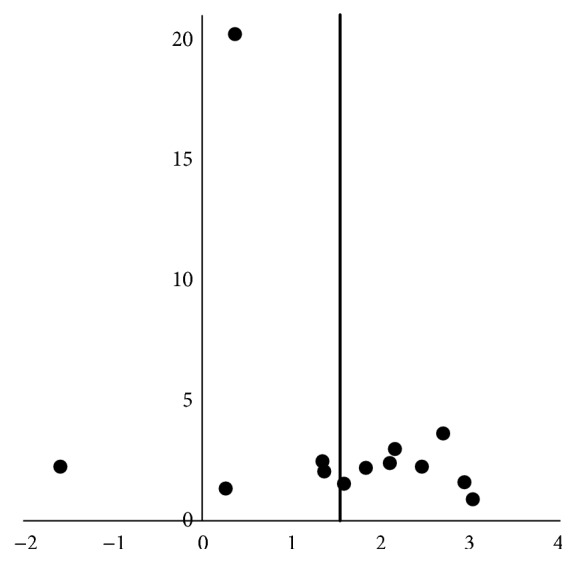
Funnel plot for publication bias. The vertical axis represents the inverse standard error. The horizontal axis represents the natural log odds ratio of the associations between poststroke anxiety and depressive symptoms. The vertical bar represents the summary estimate of odds ratios.

The asymmetrical funnel plot (Figure 3) suggests publication bias. Most published studies have shown positive association, while few studies reporting no association have been published.

### 3.2. Prestroke Anxiety and Prestroke Depression

One study [32] (*n* = 277) reported the association between prestroke anxiety and PSA and found significant correlation (OR = 2.44, *p* < 0.01). Three studies reported the association between prestroke depression and PSA; two of these found a significant correlation (OR = 2.44, *p* < 0.01) [32], (*β* = 0.64; *p* = 0.006) [30] and one [21] did not find a significant correlation (*p* = 0.05).

### 3.3. Locus of Control, Coping, and Confidence in Recovery

One study [34] stated in their methods that they would report the* Recovery Locus of Control Scale* and “recovery confidence” as psychological “predictor variables,” but these data were not reported in the study's publication. We identified no studies reporting associations between coping and PSA.

### 3.4. Fatigue

One study [19] (*n* = 98) reported associations with fatigue and found moderately strong associations between poststroke fatigue and general anxiety (*r* = 0.37, *p* < 0.001), health anxiety (*r* = 0.31, *p* < 0.01), and stroke specific anxiety (*r* = 0.37, *p* < 0.001), on univariate analysis. After hierarchical multiple regression only stroke specific anxiety remained significantly associated (*R*^2^ = 0.32, *p* < 0.001).

### 3.5. Sleep Disturbance

Two studies (*n* = 375) examined the association with sleep disturbance, both of which found an association with PSA. One [19] did not specify how sleep disturbance was measured and the other [32] used the single-item Likert Scale. We calculated an OR and 95% CI from the raw data for one (OR = 4.7, 95%  CI = 2.26–9.77, *p* ≤ 0.001) [32] and converted the regression coefficient to an OR for the other (OR = 4.05, 95%  CI = 1.87–8.79, *p* < 0.001) [19].

### 3.6. Other Psychological Associations

Four studies reported the association with cognitive impairment. Two studies [23, 26] found a significant association between cognitive impairment and PSA; one used* The Mini Mental State Examination* (MMSE) score <26 [26] (*n* = 178; OR = 0.53; 95%  CI = 0.31–0.87; *p* = 0.013); and the other used the* Victoria Stroop dots trial* which measures reduced cognitive speed [23] (*n* = 73; *r* = −0.715; *p* < 0.01); the former excluded patients with “clouding of consciousness” and moderate-severe aphasia and the latter excluded patients with aphasia. Two other studies (*n* = 2179) [22] and (*n* = 277) [32] did not find a significant association with one using the cut-off MMSE <24 [22] and the other using MMSE as a continuous variable [32]. However, the former excluded patients with severe cognitive or communicative impairment.

One study [11] reported an association between PSA and mental health related quality of life using the 12-item* Medical Outcomes Short Form Health Survey* (SF-12) and found a significant association (*n* = 535; *p* < 0.0001, effect size not available). One study [10] reported the association between PSA and trait negativity using* The Negative Affect Schedule* and found a significant association on regression analysis (*n* = 102; *β* = 0.28; *p* < 0.01). One study [27] reported the association between behavioral reactions of denial and PSA using* the Behavioral Index Form* which was found to be significantly associated (*n* = 53; *p* = 0.0065; effect size not available). The variables associated with PSA are summarised in Supplemental Table II.

## 4. Discussion

Meta-analysis of 13 studies of 2408 patients demonstrated a statistically significant association between PSA and PSD (OR = 4.66, 95%  CI = 2.23–9.74). Strikingly, there were very few studies reporting association with factors other than PSD. Very limited data suggested PSA was associated with prestroke anxiety, prestroke depression, fatigue, and sleep. Our review found no data on the associations between PSA and locus of control, coping, and confidence. Data on association between PSA and cognitive impairment were conflicting.

To our knowledge this is the only meta-analysis on the associations of PSA and psychological factors; although a systematic review was published previously [7], a meta-analysis was not performed. In addition to this, our systematic review included six further studies with 6577 patients. We performed a thorough and systematic review using a sensitive search strategy adapted from a Cochrane review. This review was based on a protocol written prior to the searches, as recommended by the Preferred Reporting Items for Systematic Reviews and Meta-Analyses Protocols (PRISMA-P) statement [38]. We used a funnel plot to assess publication bias and the STROBE quality checklist, which is endorsed as the requirement for reporting observational studies by many biomedical journals.

Our review has some limitations. Firstly, we could not include five studies; four of which were published only in abstract form and the other as a dissertation. We contacted the authors but received no response. Secondly, in our meta-analysis of the association with poststroke depressive symptoms, only three studies reported ORs that were adjusted for potential confounding factors; therefore the results from the 10 other studies included may overestimate the associations found. When we analysed the studies with adjusted and unadjusted ORs separately we found the unadjusted group had a higher summary effect than the adjusted group although there was no significant heterogeneity between the groups. There were also limitations of the individual studies: most did not report sample size calculations or describe efforts to address potential sources of bias; very few studies provided reasons for nonparticipation at each stage or explained how missing data was addressed; most studies were hospital based and 16 studies excluded patients with significant cognitive or communication impairment, limiting generalisability and making it difficult to draw conclusions on the association of PSA with cognitive impairment.

Our results and another review [4] have found PSA is common. This high prevalence supports the need to screen for anxiety in all poststroke patients. There is clear overlap between symptoms of depression and anxiety after stroke; this coexistence has been observed in other research [4] and is demonstrated by our meta-analysis. Thus in patients with depression one should look for anxiety, and vice versa. Interventions that target aspects of both anxiety and depression may also be useful. Antidepressants, for example, selective serotonin reuptake inhibitors (SSRIs), are used to treat both depression and anxiety in primary care. A randomised placebo controlled trial is the only definitive way to evaluate the effectiveness of antidepressants in poststroke anxiety and depression. The Fluoxetine or Control Under Supervision trial (ISRCTN83290762) is an ongoing UK-based multicentre randomised controlled trial of an SSRI in stroke patients. This is the largest SSRI trial ever undertaken in stroke patients and is likely to add important data to the effectiveness of pharmacological interventions for poststroke anxiety and depression.

The association we found at a single time point does not allow us to determine the direction of causality. It is plausible that another factor may cause both depression and anxiety. Therefore more longitudinal studies exploring possible direction of causality are required.

Locus of control, coping strategies, and confidence are potentially modifiable factors that could be targeted for interventions to treat anxiety after stroke. High levels of external locus of control have been found to correlate with higher levels of anxiety in patients with a generalised anxiety disorder and in patients with multiple sclerosis (MS) and ovarian cancer [39–42]. Avoidance and emotion-focused coping styles have been found to correlate with higher levels of anxiety in patients with MS [43] and specific coping strategies also correlate with higher levels of anxiety in patients with chronic obstructive pulmonary disease and lung cancer [44, 45]. Future studies need to explore the role of prestroke anxiety and depression, fatigue, sleep, locus of control, coping, and confidence to help clinicians and researchers identify those most at risk of developing PSA, in order to provide potential psychological targets to be incorporated into a psychological intervention.

## 5. Conclusions

Significant associations between anxiety and depression after stroke suggest that PSD should be screened for in patients with PSA and vice versa. The presence of anxiety or depression before stroke may be useful in identifying those most at risk of developing PSA. Other potential modifiable factors that could be targeted in interventions include fatigue and sleep disturbance. Further research into the association of PSA with potential treatment targets is required.

## Supplementary Material

The Supplementary Material comprises Table I which summarises the STROBE quality checklist for each study included in the systematic review; Table II which shows the variables found to be associated with poststroke anxiety in each study and the search strategies used to conduct the literature search.
