# Supplementary material for: Factors Associated with Poststroke Anxiety: A Systematic Review and Meta-Analysis
Source: Stroke Res Treat. 2017 Feb 22;2017:2124743. doi: 10.1155/2017/2124743 (PMC5340955; doi:10.1155/2017/2124743)
Supplement: Supplementary file 1 — The Supplementary Material comprises Table I which summarises the STROBE quality checklist for each study included in the systematic review; Table II which shows the variables found to be associated with poststroke anxiety in each study and the search strategies used to conduct the literature search. [file 2124743.f1.pdf]

ONLINE SUPPLEMENT

Psychological associations of post-stroke anxiety: A systematic review and meta-analysis

Francesca Wright, Simiao Wu MSc, Ho-Yan Yvonne Chun MRCP, Gillian Mead FRCP

STROBE Criteria			Recommendation fulfilled?																							
	Item number		Broomfield 2014	D'Aniello	Galligan	Goldfinger	Skolarus	White	Broomfield 2013	Leppavuori	Ayerbe	Fure	Kroeders	Giaquinto	Merriman	Lassalle-Lagadec	Ghika-Schmid	Sagen	Morrison	Field	Castellanos-Pinedo	Wang	Lincoln	Barker-Collo	Tang	Kim
**TITLE and ABSTRACT**	**1**	**a)**	✓	✓	✓	X	X	✓	X	X	X	X	X	X	X	X	X	X	X	✓	X	X	✓	X	X	✓
		**b)**	✓	~	✓	✓	✓	✓	✓	~	✓	~	~	✓	X	~	~	✓	~	~	~	~	~	✓	~	✓
**INTRODUCTION**																										
Background/rationale	**2**		✓	✓	✓	✓	✓	✓	✓	✓	✓	✓	✓	✓	✓	✓	✓	✓	✓	✓	✓	✓	✓	✓	~	✓
Objectives	**3**		✓	✓	✓	✓	✓	✓	✓	✓	✓	✓	✓	✓	✓	✓	~	✓	✓	✓	✓	✓	✓	✓	✓	✓
**METHODS**																										
Study design	**4**		✓	✓	✓	~	✓	✓	✓	✓	✓	✓	✓	✓	✓	✓	~	✓	✓	✓	✓	✓	✓	✓	✓	✓
Setting	**5**		✓	~	~	~	~	✓	✓	X	✓	✓	✓	✓	~	✓	X	✓	~	~	✓	✓	~	~	✓	✓
Participants	**6**	**a)**	✓	✓	✓	✓	X	✓	✓	~	~	✓	✓	✓	✓	~	X	✓	✓	✓	~	✓	✓	✓	✓	~
		**b)**	N/A	N/A	N/A	N/A	✓	N/A	N/A	N/A	N/A	N/A	N/A	N/A	N/A	N/A	N/A	N/A	N/A	N/A	N/A	N/A	N/A	N/A	N/A	N/A
Variables	**7**		~	X	~	~	~	~	~	~	✓	~	~	✓	~	X	X	~	✓	X	X	~	~	X	X	~
Data sources/measurement	**8**		✓	✓	✓	✓	✓	✓	✓	~	✓	✓	~	✓	✓	✓	✓	✓	✓	✓	✓	✓	✓	✓	✓	✓
Bias	**9**		X	X	X	X	X	X	X	X	X	X	X	X	X	X	X	X	X	X	~	X	X	X	~	X
Study size	**10**		X	X	✓	X	X	✓	X	X	X	X	X	X	X	X	X	X	X	X	X	X	X	X	X	X
Quantitive variables	**11**		✓	~	X	✓	✓	✓	✓	✓	✓	✓	✓	✓	✓	X	✓	✓	✓	~	~	✓	✓	✓	✓	✓
Statistical methods	**12**	**a)**	~	~	X	~	X	X	✓	✓	✓	✓	✓	✓	~	~	~	✓	✓	X	✓	✓	✓	~	✓	~
		**b)**	✓	~	X	X	X	✓	✓	✓	✓	N/A	N/A	N/A	N/A	~	X	N/A	✓	X	~	✓	✓	X	✓	X
		**c)**	✓	X	X	X	X	X	X	X	✓	X	X	X	X	X	X	✓	X	X	X	X	X	X	X	X
		**d)**	X	X	✓	X	X	✓	X	X	✓	X	X	X	X	X	X	X	X	X	X	X	X	X	X	X
		**e)**	X	X	X	X	✓	X	X	X	✓	X	X	X	X	X	X	X	X	X	X	X	X	X	X	X
**RESULTS**																										
Participants	**13**	**a)**	✓	X	✓	X	X	✓	✓	✓	✓	✓	✓	X	✓	X	X	✓	✓	✓	✓	✓	✓	✓	✓	✓
		**b)**	✓	X	✓	X	X	✓	✓	✓	X	✓	✓	X	✓	X	X	✓	✓	✓	X	✓	✓	X	✓	✓
		**c)**	✓	X	X	X	X	✓	X	X	✓	X	X	X	X	X	X	X	X	X	X	X	X	X	✓	X
Descriptive data	**14**	**a)**	✓	✓	✓	✓	✓	✓	X	✓	X	✓	✓	✓	✓	✓	X	✓	X	✓	✓	✓	✓	X	✓	✓
		**b)**	✓	X	X	X	✓	X	X	X	X	X	X	X	X	X	X	~	✓	✓	X	✓	✓	X	X	X
		**c)**	N/A	N/A	N/A	N/A	N/A	X	N/A	N/A	✓	N/A	N/A	N/A	N/A	✓	✓	✓	✓	✓	✓	✓	✓	N/A	N/A	X
Outcome data	**15**		✓	✓	✓	✓	✓	X	✓	✓	✓	✓	✓	✓	✓	✓	✓	✓	X	✓	✓	✓	✓	✓	✓	✓
Main results	**16**	**a)**	✓	✓	✓	✓	✓	~	✓	✓	X	✓	X	✓	X	X	X	✓	X	✓	✓	✓	✓	✓	~	~
		**b)**	✓	✓	X	✓	✓	✓	✓	N/A	✓	✓	✓	✓	✓	N/A	✓	✓	X	✓	✓	✓	✓	✓	✓	✓
		**c)**	X	X	X	X	X	X	X	X	X	X	X	X	X	X	X	X	X	X	X	X	X	X	X	X
Other analyses	**17**		X	X	X	X	✓	X	X	✓	✓	N/A	X	X	X	X	X	X	✓	X	X	~	✓	X	✓	X
**DISCUSSION**																										
Key results	**18**		✓	✓	✓	✓	✓	✓	✓	✓	✓	✓	✓	✓	✓	✓	✓	✓	✓	✓	✓	✓	✓	✓	✓	✓
Limitations	**19**		✓	✓	✓	✓	✓	✓	✓	✓	✓	✓	✓	✓	✓	✓	X	✓	✓	✓	X	✓	✓	✓	✓	✓
Interpretation	**20**		✓	✓	✓	X	✓	✓	✓	✓	✓	✓	✓	✓	✓	✓	X	✓	✓	✓	✓	✓	✓	✓	✓	✓
Generalisability	**21**		✓	~	✓	X	✓	✓	✓	✓	X	✓	✓	✓	X	✓	X	✓	✓	✓	X	✓	✓	X	✓	X
**OTHER INFORMATION**																										
Funding	**22**		X	✓	✓	X	✓	X	X	✓	✓	X	X	X	X	✓	✓	✓	✓	X	X	X	✓	X	X	X

Key

✓ = meets criteria well

~ = partially meets criteria

X = does not meet criteria well

N/A = not applicable

Table I - Table summarising STROBE quality checklist for each study.

	Variables Associated with PSA	Broomfield 2014	D'Aniello	Galligan	Goldfinger	Skolarus	White	Broomfield 2013	Leppavuori	Ayerbe	Fure	Kroeders	Giaquinto	Merriman	Lassalle-Lagadec	Ghika-Schmid	Sagen	Morrison	Field	Castellanos-Pinedo	Wang	Lincoln	Barker-Collo	Tang	Kim
**Demographic**	Older age		X				X		X		X			X			X		X			X	X	X	X
	Younger age	✓✓	X		✓✓		X	✓✓	X	✓✓	X			X			X		X			X	X	X	X
	Female	✓✓	X		✓		X	✓✓	X	✓✓	X			X				✓✓	X			X	X	X	
	Male		X						X		X			X			X		X			X	X		
	Living alone				X						✓✓														
	Socioeconomic deprivation	✓✓			✓			✓✓																	
**Pre Stroke**	Anxiety								✓											✓					
	Depression						X		✓✓			✓✓													
	Fatigue																								
**Stroke features**	Left hemisphere		X								X			X		X							✓✓		
	Right hemisphere		X						X		X			X		X								✓✓	
	Stroke severity								✓	✓✓	X											X			✓✓
	Time since stroke				X		✓							✓✓					X		X	✓✓			
	Use of anxiolytic drugs								✓✓																
	Pathological type (ischaemic/ haemorragic)																								
	Stroke subtype (TOAST or OCSP)																								
**Post Stroke**	Dementia/cognitive impairment								X	X	✓✓												✓✓		
	Disability/ADL			✓	✓✓		✓		✓	✓✓	X		✓✓	X			X				✓✓	X	X		✓✓
	Baseline anxiety						✓✓										✓✓	✓✓	✓✓		✓✓	✓✓			
	Apathy																X			✓					
	Post stroke fatigue			✓✓																					
	Post stroke depression		✓✓	✓	✓		✓✓		✓✓			✓		✓	✓		✓	✓	✓			✓	✓	✓✓	
	Sleep disturbance			✓					✓✓																
	Physical inactivity											X						X							
**Other**	Confidence																	X							
	Locus of control																	X							
	Coping																								
	Social role/participation					✓✓	✓																		
	Social support				✓✓		X																		

Table II - Variables associated with post-stroke anxiety.

Key

✓✓ = significant association found on multivariate analysis

✓= significant association found on univariate analysis

X = No significant association found

Blank = study did not look at this variable

Search Strategies

MEDLINE search strategy

1. cerebrovascular disorders/ or exp basal ganglia cerebrovascular disease/ or exp brain ischemia/ or exp carotid artery diseases/ or exp cerebrovascular accident/ or cerebrovascular trauma/ or exp hypoxia-ischaemia, brain/ or exp intracranial arterial diseases/ or exp intracranial arteriovenous malformations/ or exp "intracranial embolism and thrombosis"/ or exp intracranial hemorrhages/ or stroke/ or exp brain infarction/ or vasospasm, intracranial/ or vertebral artery dissection/
2. (stroke or poststroke or post-stroke or cerebrovasc$ or brain vasc$ or cerebral vasc$ or cva$ or apoplex$ or SAH).tw.
3. ((brain$ or cerebr$ or cerebell$ or intracran$ or intracerebral) adj5 (isch?emi$ or infarct$ or thrombo$ or emboli$ or occlus$)).tw.
4. ((brain$ or cerebr$ or cerebell$ or intracerebral or intracranial or subarachnoid) adj5 (haemorrhage$ or hemorrhage$ or haematoma$ or hematoma$ or bleed$)).tw.
5. hemiplegia/ or exp paresis/
6. (hemipleg$ or hemipar$ or paresis or paretic).tw.
7. brain injuries/ or brain injury, chronic/
8. or/1-7
9. anxiety/
10. anxiety disorders/ or agoraphobia/ or obsessive-compulsive disorder/ or panic disorder/ or phobic disorders/ or exp stress disorders, traumatic/
11. exp Anti-Anxiety Agents/
12. (anxiety or anxieties or anxious or agoraphobi$ or phobi$ or panic disorder$ or panic attack$ or (obsess$ adj3 compuls$) or post?traumatic stress$ or PTSD).tw.
13. (feel$ adj5 (apprehens$ or dread or disaster$ or fear$ or worry or worried or terror)).tw.
14. manifest anxiety scale/
15. or/9-14
16. 8 and 15

17. limit 16 to humans

EMBASE search strategy

1. cerebrovascular disease/ or basal ganglion hemorrhage/ or exp brain hematoma/ or exp brain hemorrhage/ or exp brain infarction/ or exp brain ischemia/ or exp carotid artery disease/ or cerebral artery disease/ or cerebrovascular accident/ or exp intracranial aneurysm/ or exp occlusive cerebrovascular disease/ or stroke/ or stroke patient/ or stroke unit/
2. (stroke or poststroke or post-stroke or cerebrovasc$ or brain vasc$ or cerebral vasc$ or cva$ or apoplex$ or SAH).tw.
3. ((brain$ or cerebr$ or cerebell$ or intracran$ or intracerebral) adj5 (isch?emi$ or infarct$ or thrombo$ or emboli$ or occlus$)).tw.
4. ((brain$ or cerebr$ or cerebell$ or intracerebral or intracranial or subarachnoid) adj5 (haemorrhage$ or hemorrhage$ or haematoma$ or hematoma$ or bleed$)).tw.
5. paralysis/ or hemiparesis/ or hemiplegia/ or paresis/
6. (hemipleg$ or hemipar$ or paresis or paretic).tw.
7. brain injury/
8. or/1-7
9. anxiety/
10. exp anxiety disorder/
11. exp anxiolytic agent/
12. (anxiety or anxieties or anxious or agoraphobi$ or phobi$ or panic disorder$ or panic attack$ or (obsess$ adj3 compuls$) or post?traumatic stress$ or PTSD).tw.
13. (feel$ adj5 (apprehens$ or dread or disaster$ or fear$ or worry or worried or terror)).tw.
14. beck anxiety inventory/ or hamilton anxiety scale/ or "hospital anxiety and depression scale"/ or self-rating anxiety scale/ or state trait anxiety inventory/
15. or/9-14

16. 8 and 15

17. limit 16 to human

PhyscINFO search strategy

1. cerebrovascular disorders/ or cerebral hemorrhage/ or exp cerebral ischemia/ or cerebral small vessel disease/ or cerebrovascular accidents/ or subarachnoid hemorrhage/
2. (stroke or poststroke or post-stroke or cerebrovasc$ or brain vasc$ or cerebral vasc$ or cva$ or apoplex$ or SAH).tw.
3. ((brain$ or cerebr$ or cerebell$ or intracran$ or intracerebral) adj5 (isch?emi$ or infarct$ or thrombo$ or emboli$ or occlus$)).tw.
4. ((brain$ or cerebr$ or cerebell$ or intracerebral or intracranial or subarachnoid) adj5 (haemorrhage$ or hemorrhage$ or haematoma$ or hematoma$ or bleed$)).tw.
5. hemiparesis/ or hemiplegia/
6. (hemipleg$ or hemipar$ or paresis or paretic).tw.
7. brain injur$.tw.
8. or/1-7
9. exp anxiety/
10. exp anxiety disorders/ or panic/ or panic attack/ or fear/
11. anxiety management/
12. state trait anxiety inventory/ or taylor manifest anxiety scale/
13. (anxiety or anxieties or anxious or agoraphobi$ or phobi$ or panic disorder$ or panic attack$ or (obsess$ adj3 compuls$) or post?traumatic stress$ or PTSD).tw.
14. (feel$ adj5 (apprehens$ or dread or disaster$ or fear$ or worry or worried or terror)).tw.

15. or/9-14

16. 8 and 15

CINAHL PLUS (Cumulative Index to Nursing and Allied Health Literature) search strategy

S1. cerebrovascular disorders/ or exp carotid artery diseases/ or cerebral aneurysm/ or “cerebral embolism and thrombosis”/ or exp cerebral ischemia/ or cerebral vascular accident/ or cerebral vasospasm/ or exp intracranial hemorrhage/ or vertebral artery dissections/

S2. stroke patients/ or stroke units/

S3. TI stroke or poststroke or post-stroke or cerebrovasc$ or brain vasc$ or cerebral vasc$ or cva$ or apoplex$ or SAH

S4. TI ((brain$ or cerebr$ or cerebell$ or intracran$ or intracerebral) AND (isch?emi$ or infarct$ or thrombo$ or emboli$ or occlus$))

S5. TI ((brain$ or cerebr$ or cerebell$ or intracerebral or intracranial or subarachnoid) AND (haemorrhage$ or hemorrhage$ or haematoma$ or hematoma$ or bleed$))

S6. hemiplegia/

S7. TI hemipleg$ or hemipar$ or paresis or paretic

S8. S1 or S2 or S3 or S4 or S5 or S6 or S7

S9. exp anxiety/

S9. anxiety disorders/ or generalized anxiety disorder/ or obsessive-compulsive disorder/ or panic disorder/ or social anxiety disorders/ or stress disorders, post-traumatic/ or phobic disorders/ or trichotillomania/ or body dysmorphic disorder/ or obsessive hoarding/ or agoraphobia/ or claustrophobia/

S10. "anxiety (Saba CCC)"/ or "fear (Saba CCC)"/ or self-rating anxiety scale/ or state trait anxiety inventory/ or death anxiety scale/ or "anxiety reduction (Iowa NIC)"/ or "anxiety (NANDA)"/ or "fear (NANDA)"/ or exp antianxiety agents/

S11. (anxiety or anxieties or anxious or agoraphobi$ or phobi$ or panic disorder$ or panic attack$ or (obsess$ adj3 compuls$) or post?traumatic stress$ or PTSD).tw.

S12. (feel$ adj5 (apprehens$ or dread or disaster$ or fear$ or worry or worried or terror)).tw.

S13. S9 or S10 or S11 or S12

S14. S8 and S13

AMED (Allied and Alternative Medicine) search strategy

1. cerebrovascular disorders/ or cerebral hemorrhage/ or cerebral infarction/ or cerebral ischemia/ or cerebrovascular accident/
2. (stroke or poststroke or post-stroke or cerebrovasc$ or brain vasc$ or cerebral vasc$ or cva$ or apoplexy$ or SAH).tw.
3. ((brain$ or cerebr$ or cerebell$ or intracran$ or intracerebral) adj5 (isch?emi$ or infarct$ or thrombo$ or emboli$ or occlus$)).tw.
4. ((brain$ or cerebr$ or cerebell$ or intracerebral or intracranial or subarachnoid) adj5 (haemorrhage$ or hemorrhage$ or haematoma$ or bleed$)).tw.
5. hemiplegia/
6. (hemipleg$ or hemipar$ or paresis or paretic).tw.
7. 1 or 2 or 3 or 4 or 5 or 6
8. exp anxiety/ or fear/ or panic/ or exp anxiety disorders/
9. (anxiety or anxieties or anxious or agoraphobi$ or phobi$ or panic disorder$ or panic attack$ or (obsess$ adj3 compuls$) or post?traumatic stress$ or PTSD).tw.
10. (feel$ adj5 (apprehens$ or dread or disaster$ or fear$ or worry or worried or terror)).tw.
11. 8 or 9 or 10
12. 7 and 11
